# HAtt-Flow: Hierarchical Attention-Flow Mechanism for Group-Activity Scene Graph Generation in Videos

**DOI:** 10.3390/s24113372

**Published:** 2024-05-24

**Authors:** Naga Venkata Sai Raviteja Chappa, Pha Nguyen, Thi Hoang Ngan Le, Page Daniel Dobbs, Khoa Luu

**Affiliations:** 1 Department of EECS, University of Arkansas, Fayetteville, AR 72701, USA; nchappa@uark.edu (N.V.S.R.C.); thile@uark.edu (T.H.N.L.); 2 Department of Health, Human Performance and Recreation, University of Arkansas, Fayetteville, AR 72701, USA; pdobbs@uark.edu

**Keywords:** group activity recognition, flow-attention, video scene graph

## Abstract

Group-activity scene graph (GASG) generation is a challenging task in computer vision, aiming to anticipate and describe relationships between subjects and objects in video sequences. Traditional video scene graph generation (VidSGG) methods focus on retrospective analysis, limiting their predictive capabilities. To enrich the scene-understanding capabilities, we introduced a GASG dataset extending the JRDB dataset with nuanced annotations involving *appearance, interaction, position, relationship, and situation* attributes. This work also introduces an innovative approach, a **H**ierarchical **Att**ention–**Flow** (HAtt-Flow) mechanism, rooted in flow network theory to enhance GASG performance. Flow–attention incorporates flow conservation principles, fostering competition for sources and allocation for sinks, effectively preventing the generation of trivial attention. Our proposed approach offers a unique perspective on attention mechanisms, where conventional “values” and “keys” are transformed into sources and sinks, respectively, creating a novel framework for attention-based models. Through extensive experiments, we demonstrate the effectiveness of our Hatt-Flow model and the superiority of our proposed flow–attention mechanism. This work represents a significant advancement in predictive video scene understanding, providing valuable insights and techniques for applications that require real-time relationship prediction in video data.

## 1. Introduction

Visual scene understanding is a foundational challenge in computer vision, encompassing the interpretation of complex scenes, objects, and their relationships within images and videos. This task is particularly intricate for video, where temporal dynamics and multi-modal information introduce unique complexities. Group-activity video scene graph (GAVSG) generation, which involves predicting relationships between objects in a video across multiple frames, stands at the forefront of this endeavor.

In recent years, significant progress has been made in video understanding. Techniques such as video scene graph generation (VidSGG) have allowed us to extract high-level semantic representations from video content. However, VidSGG typically operates in a static, retrospective manner, constraining its predictive capabilities. The GAVSG dataset, on the other hand, extends the scope of visual scene understanding to anticipate and describe subject-and-object relationships and their temporal evolution.

In the closely linked domain of human–object interaction (HOI) [1], transferable techniques have proven effective for scene graph generation (SGG) tasks [2,3,4,5,6,7,8,9,10,11,12,13,14,15,16,17,18], some of which inspired the foundation of PSG dataset baselines [19,20].

**Figure 1:**
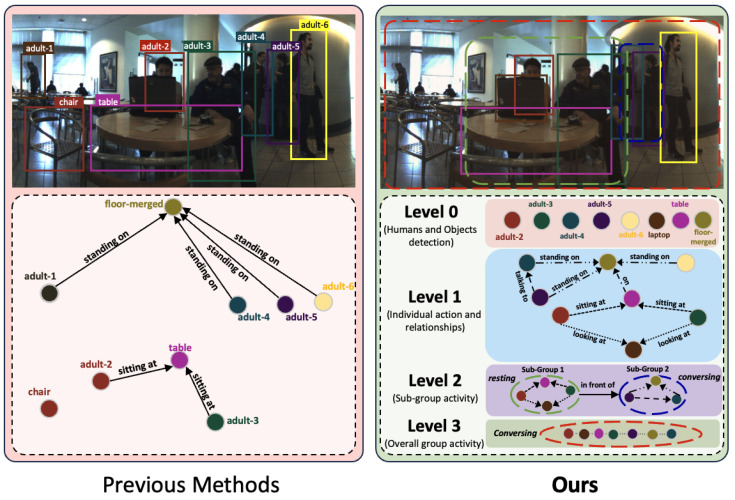
Comparison of HAtt-Flow results with other scene graph generation methods. **Best viewed in color and zoomed in.**

In the context of SGG, diverse methodologies have been explored, from probabilistic graphical models and AND-OR grammar approaches [21,22,23,24,25,26,27,28] to knowledge graph embeddings like VTransE [29] and UVTransE [30]. Recent endeavors delve into challenges such as the long-tailed distribution of predicates [31,32], visually irrelevant predicates [33], and precise bounding box localization [34]. As shown in Figure 1, we can observe that the previous methods can detect the subjects and objects in a scene. However, they need to generate a well-defined scene graph, whereas our method can learn all the nuanced relationships among the subjects and objects in the scene to produce a fine scene graph.

To address the limitation of the enriched learning of group activities in a scene, we introduced the GASG dataset, which includes nuanced annotations in the form of five different attributes. This can help set a better scene graph generation benchmark than the existing datasets in this domain. In this work, we propose a novel approach for GAVSG that draws inspiration from flow network theory, introducing flow–attention. This mechanism leverages flow conservation principles in both the source and sink aspects, introducing a competitive mechanism for sources and an allocation mechanism for sinks. This innovative approach mitigates the generation of trivial attention and enhances the predictive power of GAVSG. We build upon a new perspective on attention mechanisms, rooted in flow network theory, to design our GAVSG framework. The conventional attention mechanism aggregates information from “values” and “keys” based on the similarity between “queries”. By framing attention in terms of flow networks, we transform values into sources and keys into endpoints, thus creating a fresh perspective on the attention mechanism.

**Contributions:** The main contributions of our work are threefold. **First**, we introduce a novel dataset (the dataset is available for verification at https://uark-cviu.github.io/GASG/) with nuanced attributes that aid the scene graph generation task in the group activity setting. **Second**, our work advances the state of the art in predictive video scene understanding by introducing flow–attention and redefining attention mechanisms via incorporating hierarchy awareness. **Third**, we demonstrate the effectiveness of our approach through extensive experiments and achieve state-of-the-art performance over the existing approaches.

## 2. Related Work

**Group action recognition (GAR).** Group action recognition (GAR) has witnessed a shift towards deep learning methodologies, notably convolutional neural networks (CNNs) and recurrent neural networks (RNNs) [35,36,37,38,39,40,41,42,43,44,45]. Attention-based models and graph convolution networks are crucial in capturing spatial–temporal relations in group activities. Transformer-based encoders, often coupled with diverse backbone networks, excel in extracting features for discerning actor interactions in multimodal data [46]. Recent innovations, such as MAC-Loss, introduce dual spatial and temporal transformers for enhanced actor interaction learning [47]. The field continues to evolve with heuristic-free approaches like those by Tamura et al., simplifying the process of social group activity recognition and member identification [48].

**Scene graph generation (SGG).** In scene graph generation (SGG), the traditional two-stage paradigm involves object detection and pairwise predicate estimation [49,50,51,52,53,54,55,56,57,58,59,60]. Recent advancements include knowledge graph embeddings, graph-based architectures, energy-based models, and linguistic supervision [56,61,62,63,64,65,66,67,68,69]. To address challenges like long-tailed distribution and visually irrelevant predicates, the field has seen a pivot towards panoptic segmentation-based SGG, inspired by the simultaneous generation of scene graphs and semantic segmentation masks [34]. Notably, insights from the closely linked domain of human–object interaction (HOI) have influenced SGG techniques [2,3,4,5,6,7,8,9,10,11,12,13,14,15,16,17,18,70,71].

**Video scene graph generation (VidSGG).** VidSGG, initiated by Shang et al. [72], explores spatio-temporal relations in videos. Research has delved into spatio-temporal conditional bias, the domain shift between image and video scene graphs, and embodied semantic approaches using intelligent agents. Notable methods include TRACE [73], which separates relation prediction and context modeling, and embodied semantic SGG, employing reinforcement learning for path generation via intelligent agents [74,75]. Yang et al. [76] proposed a transformer–encoder-based baseline model to evaluate their proposed panoptic video scene graph dataset, which included fusing the extracted features of the subjects in the scene.

### Limitation of Prior Datasets

We present a detailed comparison of existing datasets in Table 1. However, upon further examination, it becomes apparent that the limitations of prior datasets are particularly pronounced when considering the intricate nature of group activities within visual content. Many existing datasets have primarily focused on specific types of individual actions, often overlooking the complex dynamics of group activities in real-world interactions. This narrow focus has hindered the development of models capable of addressing a diverse range of action classifications, thereby limiting their adaptability to varied real-world  scenarios.

Moreover, earlier datasets have often provided sparse annotations and underscored relationships within isolated fragments of the relational graph, neglecting the complexities of broader and more intricate scenes. This sparse annotation may lead to a lack of comprehensive relationship modeling and potential biases in the developed models.

Furthermore, there exists a significant gap in the representation of scenes featuring dense crowds of people. These settings present formidable challenges related to occlusion management and require a nuanced understanding of complex interactions within such contexts.

In response to these identified limitations, we introduce the *group activity scene graph* (GASG) dataset. This dataset directly addresses the aforementioned issues by featuring various scenes and settings (represented as attributes in the annotations), spanning distinct scenarios and effectively differentiating it from prior datasets. The GASG dataset excels in capturing five critical features of group activities: appearance, situation, position, interaction, and relations. Additionally, it comprehensively tracks the movements and interactions of individuals and sub-groups, facilitating a profound understanding of their dynamics and activities over time. With its rich dataset of these aspects, the GASG dataset lays the groundwork for a new paradigm in understanding complex group activities within scenarios characterized by dense populations, thereby pushing the boundaries of group activity recognition.

## 3. Dataset Overview

The GASG dataset offers a diverse array of sub-group and group activities, enriching the landscape of research in group activity recognition. It encompasses 27 categories of individual actions, such as walking and talking, aligning with the categories present in the JRDB-Act dataset. Additionally, our dataset presents 11 distinct categories of sub-group activities, ranging from standing closely and chatting to complex interactions like group evolution and collaborative work. These sub-group activities meticulously capture the nuances of interpersonal dynamics within smaller groups, providing a nuanced portrayal of real-world interactions.

Furthermore, the dataset encapsulates seven categories of group activities, including walking, conversing, commuting, resting, office working, waiting, and sitting. These activities encapsulate commonplace scenarios encountered in real-world settings, serving as a robust foundation for analyzing collective behaviors and interactions within larger groups. By incorporating a comprehensive spectrum of activities, the GASG dataset empowers researchers to delve deeply into the complexities of group dynamics and advance the frontiers of group activity recognition.

### 3.1. Data Collection and Annotation

This GASG dataset comprises a rich collection of videos from the JRDB dataset, offering a unique perspective on sub-group and overall group activities. This dataset provides comprehensive coverage of various activities and introduces essential tracking information.

**Figure 2:**
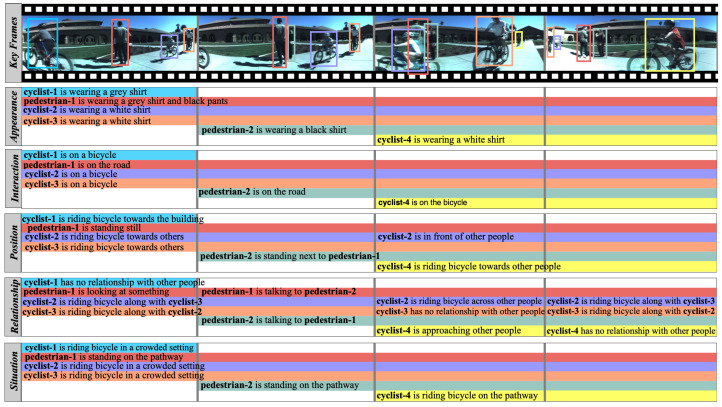
**A sample video from our group activity scene graph (GASG) dataset.** The top row displays keyframes featuring overlaid bounding boxes, each annotated with a unique ID for consistency. Below, the timeline tubes provide a comprehensive temporal representation of scene graph annotations for distinct attributes, including ***appearance, interaction, position, relationship, and situation***. These annotations offer nuanced details, enhancing scene understanding and contributing to a more refined video content analysis. **Best viewed in color and zoomed in.**

The tracking information within the GASG dataset (as shown in Figure 2) facilitates a detailed understanding of individuals and sub-groups within each frame. This information includes the trajectory, position, and interactions of each actor. The dataset defines five key aspects for comprehensive scene understanding:

- *Interaction*: This aspect characterizes the dynamic interactions between subjects and objects, shedding light on how individuals and sub-groups engage with each other.
- *Position*: The dataset includes precise data on the location and orientation of subjects and objects, enhancing the analysis of their spatial relationships during activities.
- *Appearance*: Visual traits of subjects and objects are meticulously captured, allowing for detailed examinations of their attributes and characteristics.
- *Relationship*: Understanding the associations and connections between subjects and objects is essential for deciphering the complex interplay within group activities. This aspect provides insight into the underlying dynamics of relationships within the scenes.
- *Situation*: To provide environmental context, the GASG dataset offers descriptors highlighting the contextual information surrounding subjects and objects, enabling researchers to consider the broader setting in their analyses.

These five key aspects—interaction, position, appearance, relationship, and situation—form the backbone of the dataset’s annotation structure, providing a holistic view of the diverse activities and interactions within the sub-group and overall group scenarios. This level of detail sets GASG apart, making it a valuable resource for research in scene comprehension, action recognition, and group activity analysis. The annotation process is detailed in the Appendix B.

### 3.2. Dataset Statistics

**Figure 3:**
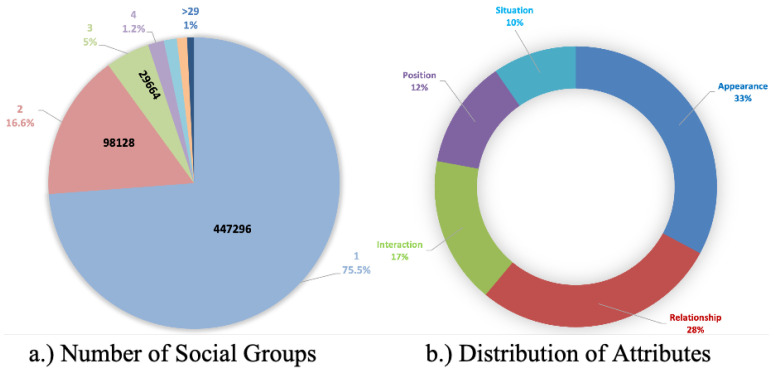
Statistics of the GASG dataset, number of social groups, and attributes in the dataset. **Best viewed in color and zoomed in.**

The accompanying pie chart in Figure 3b delves into the complexity of attributes in our dataset, revealing that “Appearance” consists of 33% of the total annotations, which is the highest, “Relationship” consists of 28%, “Interaction” consists of 17%, “Position“ consists of 12%, and “Situation” consists of 10%, which is the least. We explore the distribution of social activity labels in Figure 3a, focusing on the sizes of social groups. The chart in the figure provides a nuanced view of social group sizes in the dataset. Specifically, 75.5%, 16.6%, 5%, and 1.2% of social groups consist of one, two, three, and four members, respectively. Interestingly, only 1% of the dataset includes groups with five or more members, with the maximum observed group size being 29 members.

## 4. Methodology

In this section, we present the methodology of our proposed HAtt-Flow approach for robust group activity recognition. We introduce three key modules: input preparation, hierarchical awareness induction, and feature flow–attention mechanism, which are overviewed below:

**Input preparation**: We prepare input node and edge embeddings for the graph transformer layer. This module uniquely incorporates both textual and visual features, facilitating a holistic representation of the underlying data. The novel aspect here is the integration of both modalities into a unified representation, enabling the model to leverage complementary information from both sources for improved recognition accuracy.

**Hierarchical awareness induction**: We propose enriching the vision and language branches through a novel hierarchy-aware attention mechanism. This module introduces hierarchical aggregation priors to guide the model in capturing complex relationships within the data. The novelty lies in integrating hierarchical information into the attention mechanism, allowing the model to capture multi-level dependencies and semantic hierarchies within group activities.

**Feature flow-attention mechanism**: Inspired by flow network theory, we introduce a feature flow–attention mechanism to prevent the generation of trivial attention s. This module incorporates competitive and allocation principles to enhance the model’s ability to capture relevant features within group activities. The innovation here is the introduction of a flow-based attention mechanism, which enables the model to dynamically allocate attention based on feature importance, leading to more robust and interpretable representations of group activities.

Additionally, we present our training loss formulation tailored for the HAtt-Flow architecture, which encourages the joint learning of textual and visual features for improved group activity recognition.

**Figure 4:**
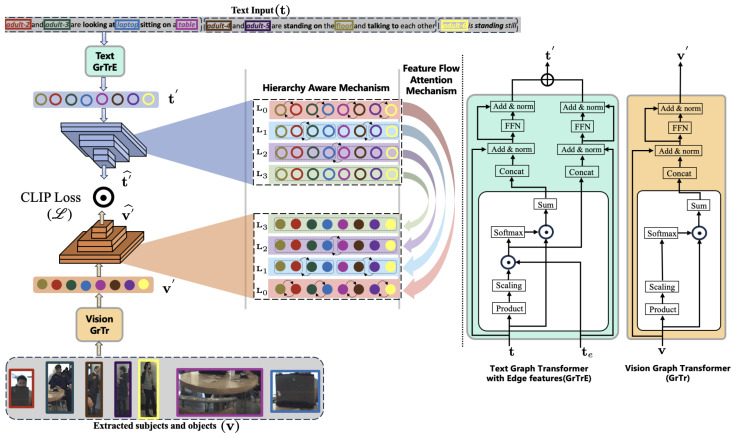
**Overall architecture of the proposed HAtt-Flow network.** The extracted visual and textual features are passed through their respective graph transformers to obtain corresponding node features. These nodes are passed through the hierarchy-aware-based transformer encoder models to have enriched features, including a feature flow–attention mechanism to enhance cross-modality learning. Finally, we use CLIP loss to optimize the learned features. Please refer to Figure 1 for the details of levels $L_{0}$, $L_{1}$, $L_{2}$, and $L_{3}$.

We utilized pre-trained visual and textual backbones to extract the corresponding subject features in the video, v, and textual features, t. In the input preparation section, these are *h* to represent the nodes and *e* to represent the edges. However, we denote visual nodes as v, textual nodes as t, and textual edges as $t_{e}$ in Figure 4. We used the graph transformer layer and the graph transformer layer with edge features to extract the corresponding feature representations. The former is tailored to graphs lacking explicit edge attributes, while the latter incorporates a dedicated edge feature pipeline to integrate available edge information, maintaining abstract representations at each layer.

Now, let us proceed to detail each module in the subsequent subsections.

### 4.1. Input Preparation

Initially, we prepared input node and edge embeddings for the graph transformer layer. In the context of our model, text features are employed to generate both nodes and edges, whereas vision features are exclusively utilized for generating nodes. Consider a graph, G, with node features represented as text features, $\alpha_{i}\in R^{d_{n}\times 1}$, for each node, *i*, and edge features, also derived from text, denoted as $\beta_{ij}\in R^{d_{e}\times 1}$ for edges between nodes *i* and *j*. The input node features, $\alpha_{i}$, and edge features, $\beta_{ij}$, undergo a linear projection to be embedded into *d*-dimensional hidden features, $h_{i}^{0}$ and $e_{ij}^{0}$.

(1) $$\hat{h}i^{0}=A^{0}\alpha_{i}+a^{0};e{ij}^{0}=B^{0}\beta_{ij}+b^{0},$$

Here, $A^{0}\in R^{d\times d_{n}}$, $B^{0}\in R^{d\times d_{e}}$ and $a^{0},b^{0}\in R^{d}$ are parameters of the linear projection layers. The pre-computed node positional encodings of dimension *k* are linearly projected and added to the node features ${\hat{h}}_{i}^{0}$.

(2) $$\lambda_{i}^{0}=C^{0}\lambda_{i}+c^{0};h_{i}^{0}={\hat{h}}_{i}^{0}+\lambda_{i}^{0},$$

Here, $C^{0}\in R^{d\times k}$, and $c^{0}\in R^{d}$. Notably, positional encodings are only added to the node features at the input layer and not during intermediate graph transformer layers. Detailed information about the graph transformer layers is presented in the Appendix C.

### 4.2. Hierarchical Awareness Induction

We propose enriching the vision and language branches through a hierarchy-aware attention mechanism. In line with the conventional transformer architecture, we divide modality inputs into low-level video patches and text tokens. These are recursively merged based on semantic and spatial similarities, gradually forming more semantically concentrated clusters, such as video objects and text phrases. We define hierarchy aggregation priors with the following aspects:

**Tendency to merge.** Patches and tokens are recursively merged into higher-level clusters that are *spatially and semantically* similar. If two nearby video patches share similar appearances, merging them is a natural step to convey the same semantic information.

**Non-splittable.** Once patches or tokens are merged, they will *never be split* in later layers. This constraint ensures that hierarchical information aggregation never degrades, preserving the complete process of hierarchy evolution layer by layer.

We incorporate these hierarchy aggregation priors into an attention mask, *C*, serving as an extra inductive bias to help the conventional attention mechanism in transformers better explore hierarchical structures adapted to each modality format—a 2D grid for videos and a 1D sequence for texts. Thus, the proposed hierarchy-aware attention is defined as follows:

(3) $$Hierarchy\_Attention=\left(C⊙\mathrm{softmax}\left(\frac{QK^{T}}{\sqrt{d_{h}}}\right)\right)V$$

Note that *C* is shared among all heads and progressively updated bottom-up across transformer layers. We elaborate on the formulations of the hierarchy-aware mask, *C*, for each modality as follows.

**Hierarchy Induction for Language Branch**

In this section, we reconsider the tree-transformer method from the perspective of the proposed hierarchy-aware attention, explaining how to impose hierarchy aggregation priors on *C* in three steps.

*Generate neighboring attention score.* The merging tendency of adjacent word tokens is described through neighboring attention scores. Two learnable key and query matrices, $W_{Q}^{\prime }$ and $W_{K}^{\prime }$, transfer any adjacent word tokens, $\left(t_{i},t_{i+1}\right)$. The neighboring attention score, $s_{i,i+1}$, is defined as their inner product:

(4) $$s_{i,i+1}=\frac{\left(t_{i}W_{Q}^{\prime }\right)\cdot \left(t_{i+1}W_{K}^{\prime }\right)}{\sigma_{t}}$$

Here, $\sigma_{t}$ is a hyperparameter controlling the scale of the generated scores. A softmax function for each token, $t_{i}$, is employed to normalize its merging tendency with two neighbors:

(5) $$p_{i,i+1},p_{i,i-1}=\mathrm{softmax}\left(s_{i,i+1},s_{i,i-1}\right)$$

For neighbor pairs $\left(t_{i},t_{i+1}\right)$, the neighboring affinity score ${\hat{a}}_{i,i+1}$ is the geometric mean of $p_{i,i+1}$ and $p_{i+1,i}$: ${\hat{a}}_{i,i+1}=\sqrt{p_{i,i+1}\cdot p_{i+1,i}}$. From a graph perspective, it describes the strength of edge $e_{i,i+1}$ by comparing it with edges $e_{i-1,i}$ ($p_{i,i+1}\;\mathrm{vs}.\;p_{i,i-1}$) and $e_{i+1,i+2}$ ($p_{i+1,i}\;\mathrm{vs}.\;p_{i+1,i+2}$).

*Enforcing non-splittable property.* A higher neighboring affinity score indicates that two neighbor tokens are more closely bonded. To ensure that merged tokens will not be split, layer-wise affinity scores, $a_{i,i+1}^{l}$, should increase as the network goes deeper, i.e., $a_{i,i+1}^{l}\geq a_{i,i+1}^{l-1}$ for all *l*. It helps to gradually generate a desired hierarchy structure:

(6) $$a_{i,i+1}^{l}=a_{i,i+1}^{l-1}+\left(1-a_{i,i+1}^{l-1}\right){\hat{a}}_{i,i+1}^{l}$$

Similarly, we formulate the *hierarchy induction for visual branch*, detailed in the Appendix D.

### 4.3. Feature Flow–Attention Mechanism

In the following representation, we use the corresponding nodes, *h*, from the respective branches of language and visual graph transformers as the queries (Q), keys (K), and values (V).

Inspired by flow network theory, the flow–attention mechanism introduces a competitive mechanism for sources and an allocation mechanism for sinks, preventing the generation of trivial attention. In a flow network framework, attention is viewed as the flow of information from sources to sinks. The results (R) act as endpoints receiving the inbound information flow, and the values (V) serve as sources providing the outgoing information flow.

**Flow capacity calculation.** For a scenario with ***n*** sinks and ***m*** sources, incoming flow, $I_{i}$, for the *i*-th sink and outgoing flow, $O_{j}$, for the *j*-th source are calculated as follows:

(7) $$\begin{array}{cc} I_{i} & =\phi \left(Q_{i}\right)\sum_{j=1}^{m}\phi {\left(K_{j}\right)}^{T},\\ O_{j} & =\phi \left(K_{j}\right)\sum {i=1}^{n}\phi {\left(Q_{i}\right)}^{T}, \end{array}$$

where ϕ(·) is a non-negative function.

**Flow conservation.** We establish the preservation of incoming flow capacity for each sink, maintaining the default value at 1, effectively “locking in” the information forwarded to the next layer. This conservation strategy ensures that the outgoing flow capacities of sources engage in competition, with their collective sum strictly constrained to 1. Likewise, by conserving the outgoing flow capacity for each source at the default value of 1, essentially “fixing” the information acquired from the previous layer, the conservation of incoming and outgoing flow capacities is enforced via normalizing operations:

(8) $$\begin{array}{c} \frac{\phi (K)}{O},\frac{\phi (Q)}{I}, \end{array}$$

In this context, the ratio denotes element-wise division, with $\frac{\phi (K)}{O}$ dedicated to source conservation and $\frac{\phi (Q)}{I}$ assigned to sink conservation.

This normalization process ensures the preservation of flow capacity for each source and sink token, as evidenced by the following equations:

(9) $$\begin{array}{cc} \mathrm{source}-j:\; & \frac{\phi {\left(K_{j}\right)}^{T}}{O_{j}}\sum_{i=1}^{n}\phi \left(Q_{i}\right)=\frac{\sum_{i=1}^{n}\phi \left(Q_{i}\right)\phi {\left(K_{j}\right)}^{T}}{O_{j}}=1\\ \sin k-i:\; & \frac{\phi {\left(Q_{i}\right)}^{T}}{I_{i}}\sum_{j=1}^{m}\phi \left(K_{j}\right)=\frac{\sum_{j=1}^{m}\phi \left(K_{j}\right)\phi {\left(Q_{i}\right)}^{T}}{I_{i}}=1 \end{array}$$

These equations replicate the same computations as Equation (7). The initial equation concerns the outgoing flow capacity of the *j*-th source after the normalization process $\frac{\phi (K)}{O}$. In contrast, the second equation corresponds to the incoming flow capacity of the *i*-th sink after the normalization process $\frac{\phi (Q)}{I}$. In both instances, the capacities are identical to the default value of 1.

The conserved incoming flow, $\hat{I}$, and outgoing flow, $\hat{O}$, are represented as follows:

(10) $$\begin{array}{cc} \hat{I} & =\phi \left(Q\right)\sum_{j=1}^{m}\frac{\phi {\left(Kj\right)}^{T}}{Oj},\\ \hat{O} & =\phi \left(K\right)\sum_{i=1}^{n}\frac{\phi {\left(Qi\right)}^{T}}{Ii} \end{array}$$

**Flow–attention mechanism.** We introduce the flow–attention mechanism, leveraging competition induced via incoming flow conservation for sinks. In $\hat{O}$, sources compete while maintaining a fixed flow-capacity sum, revealing source significance. $\hat{I}$ represents the sink information when the source outgoing capacity is 1, reflecting aggregated information allocation to each sink. The flow–attention equations are as follows:

(11) $$\begin{array}{cc} \mathrm{Competition}:\; & \hat{V}=\mathrm{Softmax}\left(\hat{O}\right)⊙V\\ \mathrm{Aggregation}:\; & A=\frac{\phi (Q)}{I}\left(\phi {\left(K\right)}^{T}\hat{V}\right)\\ \mathrm{Allocation}:\; & R=\mathrm{Sigmoid}(\hat{I})⊙A. \end{array}$$

In the “Competition” stage, $\hat{V}$ is determined through the application of the Softmax function to $\hat{O}$, followed by element-wise multiplication with V. The “Aggregation” step, denoted as A, is computed using the presented equation. Lastly, the “Allocation” phase calculates R by employing the Sigmoid function on $\hat{I}$, which is then element-wise multiplied with A.

### 4.4. Training Loss

To adapt the contrastive pretraining objective for video and text features in the HAtt-Flow architecture, the objective function can be expressed as follows:

(12) $$\begin{array}{c} L=-\frac{1}{M}\sum_{i}^{M}\log\frac{\exp\left(v_{i}^{⊤}u_{i}/\tau \right)}{\sum_{j=1}^{M}\exp\left(v_{i}^{⊤}u_{j}/\tau \right)}-\frac{1}{M}\sum_{i}^{M}\log\frac{\exp\left(u_{i}^{⊤}v_{i}/\tau \right)}{\sum_{j=1}^{M}\exp\left(u_{i}^{⊤}v_{j}/\tau \right)} \end{array}$$

Here, v and u represent the video and text feature vectors, τ is the learnable temperature parameter, and *M* is the total number of video–text pairs, i.e., the total number of labels.

## 5. Experimental Results

### 5.1. Experiment Settings

**Dataset details.** Our dataset adopts a division strategy from JRDB [81], where videos are segregated at the sequence level, ensuring the entirety of a video sequence is allocated to a specific split. The 54 video sequences are distributed, with 20 for training, seven for validation, and 27 for testing. To align with the evaluation practices of analogous datasets, our evaluation is centered on keyframes sampled at one-second intervals, resulting in 1419 training samples, 404 validation samples, and 1802 test samples.

**Implementation details.** Our framework, implemented in PyTorch, undergoes training on a machine featuring four NVIDIA Quadro RTX 6000 GPUs. During training, we adopt a batch size of 2 and leverage the Adam Optimizer, commencing the training process with an initial learning rate set at 0.0001.

**Evaluation metrics.** We evaluate the model using two tasks: (1.) predicate classification (PredCls) and (2.) video scene graph generation (VSGG). The video scene graph generation (VSGG) task aims to generate descriptive triplets for an input video. Each triplet, denoted as ($r_{i},t_{1},t_{2},o_{s},{m_{s}}^{\left(t_{1},t_{2}\right)},o_{o},{m_{o}}^{\left(t_{1},t_{2}\right)}$), consists of a relation, $r_{i}$, occurring between time points $t_{1}$ and $t_{2}$, connecting a subject, $o_{s}$, (class category) with mask tube ${m_{s}}^{\left(t_{1},t_{2}\right)}$ and an object, $o_{o}$, with mask tube ${m_{o}}^{\left(t_{1},t_{2}\right)}$. Evaluation metrics for PredCls and VSGG adhere to scene graph generation (SGG) standards, utilizing Recall@K (R@K) and mean Recall@K (mR@K). Successful recall for a ground-truth triplet (${\hat{o}}_{s},{{\hat{m}}_{s}}^{\left({\hat{t}}_{1},{\hat{t}}_{2}\right)},{\hat{o}}_{o},{{\hat{m}}_{o}}^{\left({\hat{t}}_{1},{\hat{t}}_{2}\right)},{{\hat{r}}_{i}}^{\left({\hat{t}}_{1},{\hat{t}}_{2}\right)}$) requires accurate category labels and IOU volumes between predicted and ground-truth mask tubes above 0.5. The soft recall is recorded when these criteria are met, considering the time IOU between predicted and ground-truth intervals.

### 5.2. Comparison with the State of the Art

We present our comparisons with state-of-the-art (SOTA) methods in Table 2 and Table 3 for our dataset and PSG dataset. In direct comparison with the methods above, the HAtt-Flow model exhibits a notable performance advantage, establishing itself as the current state of the art. This superiority is attributed to its proficiency in capturing intricate social activities among subjects across spatial and temporal dimensions. On the GASG dataset, our proposed method outperforms existing SGG methods by a significant margin on all metrics except for the R/mR@20 of the VSGG task. On the PSG dataset, it is evident that the proposed method dominated the other methods to demonstrate state-of-the-art performance.

### 5.3. Ablation Study

**Flow-attention direction.** We introduced a novel flow–attention mechanism between the hierarchical transformers handling text and vision. To explore the impact of the flow direction between these networks, we conducted experiments as detailed in Table 4. Our findings validate that optimal results are achieved when attention flows from the text to the vision transformer. Conversely, performance declines in the opposite direction, notably when no attention flows. This observation suggests that the cross-attention mechanism enhances the model’s contextual learning capacity primarily when the flow is from text to vision because text often provides high-level semantic information and context that can guide the understanding of visual content. However, it is worth noting that further investigation is warranted to fully understand the reasons behind the decline in performance when attention flows in the opposite direction. Potential improvements could involve refining the interaction between the text and vision transformers to better leverage complementary information from both modalities.

**Importance of hierarchy awareness.** We incorporated hierarchy awareness into the transformer framework to bolster the model’s scene graph generation capabilities. The experimental results, detailed in Table 5, affirm that hierarchical awareness optimally enhances scene graph generation. Conversely, performance declines in the absence of this design. This is likely because, when the model is aware of the hierarchy during the generation of video scene graphs, it accurately predicts all relevant nodes and their relationships (edges). However, future work could explore alternative methods for incorporating hierarchy awareness to further improve performance, such as fine-tuning the hierarchical structure or exploring different aggregation techniques.

**Attribute analysis.** In Table 6, we evaluate the impact of different attributes in the dataset on the model’s performance in scene graph generation. Our findings affirm that including all attributes results in optimal scene graph generation. Conversely, the performance exhibits a decline when we consider individual attributes one by one. We can clearly observe that performance is proportional to the attribute distribution, as shown in Table 3, i.e., the more the number of attributes, the better performance. This underscores the significance of leveraging all attributes, indicating that they collectively enhance the model’s capacity to grasp intricate contexts, enabling accurate scene graph generation. However, further investigation into the interplay between different attributes and their impact on performance could provide valuable insights for refining the model architecture and training process.

## 6. Qualitative Analysis

**Figure 5:**
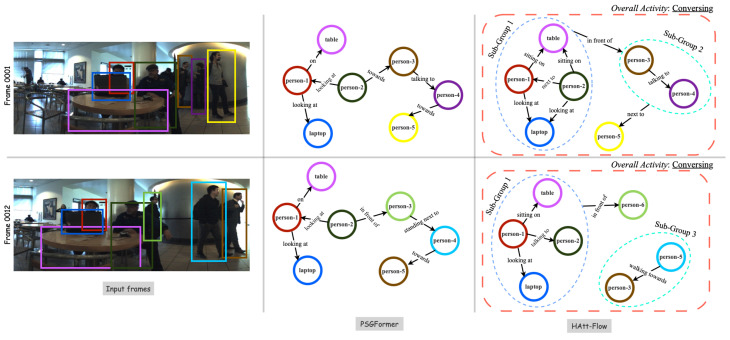
**The visualization of the scene graphs generated via PSGFormer [80] and our approach.** We can observe that [80] could only detect the subjects, but not accurate groups and their interactions. In contrast, the HAtt-Flow is accurate in graph generation and overall group activity prediction. **Best viewed in color and zoomed in.**

To gain deeper insights into the performance of HAtt-Flow, we employed visualization to illustrate its scene graph generation predictions using our dataset. As depicted in Figure 5, our model substantially improves overall scene graph generation compared to PSGFormer. The effectiveness of our hierarchy-aware attention–flow mechanism contributes significantly to this enhancement, providing our model with superior context modeling capabilities for visual representations guided by textual inputs.

## 7. Conclusions

In this work, we introduced a pioneering dataset designed with nuanced attributes, specifically tailored to enhance the scene graph generation task within the context of group activities. Our contributions extend to advancing predictive video scene understanding, propelled by the introduction of flow–attention and a paradigm shift in attention mechanisms through hierarchy awareness. Via rigorous experimentation, we demonstrated the efficacy of our approach, showcasing significant improvements over the previously existing methods.

### Limitations

While the novel flow–attention mechanism introduced in this work draws inspiration from flow network theory and offers promising advancements, several limitations warrant consideration. Primarily, the implementation of flow–attention imposes heightened computational demands, which may pose challenges for deployment in resource-constrained settings. This computational complexity underscores the need for efficient algorithms and hardware acceleration techniques to enable practical use in real-world applications. Furthermore, the performance of the flow–attention model is greatly influenced by the quality and quantity of available training data. Variations in data quality or insufficient sample sizes can impact the model’s robustness and generalization capabilities, highlighting the importance of extensive and diverse datasets for achieving optimal results. Addressing these computational and data-related limitations is crucial to maximizing the practicality and effectiveness of the proposed flow–attention mechanism. Future research endeavors should focus on developing strategies to mitigate computational demands while maintaining performance levels and exploring methods for enhancing the robustness of the model to variations in training data. By overcoming these challenges, the flow–attention mechanism can realize its full potential as a valuable tool in a wide range of real-world applications.

## Figures and Tables

**Table 1:** Comparison of existing datasets. **GA** is the group activity label, **H-H**, **H-O**, and **O-O** represent interactions between *human and human, human and object, and object and object*.

Datasets	Settings	Annotations			Attributes		
		BBox	IDs	GA	H-H	H-O	O-O
ActivityNet [77]	1	✓	✗	✗	✗	✓	✗
MSRVTT [78]	1	✓	✗	✗	✗	✓	✗
MSVD [79]	1	✓	✗	✗	✗	✓	✓
PSG [80]	1	✓	✓	✗	✓	✓	✓
PVSG [76]	1	✓	✓	✗	✓	✓	✓
**GASG (Ours)**	**5**	✓	✓	✓	✓	✓	✓

**Table 2:** Comparison with SOTA methods on **GASG dataset**. Bold numbers indicate the best results.

Method	Modality (X)	R/mR@20		R/mR@50		R/mR@100	
		PredCls	VSGG	PredCls	VSGG	PredCls	VSGG
IMP [54]	Image	31.9/9.55	16.5/6.52	36.8/10.9	18.2/7.05	38.9/11.6	18.6/7.23
	Video	-	-	-	-	-	-
MOTIFS [55]	Image	44.9/20.2	20.0/9.10	50.4/20.1	21.7/9.57	52.4/22.9	22.0/9.67
	Video	-	-	-	-	-	-
VCTree [56]	Image	45.3/20.7	20.6/9.70	50.8/22.6	22.1/10.2	52.7/23.3	22.5/10.2
	Video	-	-	-	-	-	-
GPSNet [58]	Image	31.5/13.2	17.8/2.03	39.9/16.4	19.6/7.49	44.7/18.3	20.1/7.67
	Video	-	-	-	-	-	-
PSG [80]	Image	-	31.4/16.2	-	32.9/21.5	-	36.1/22.7
	Video	-	-	-	-	-	-
PVSG [76]	Image	-	38.3/18.1	-	41.7/20.8	-	43.2/23.7
	Video	-	**13.6/10.2**	-	19.2/11.1	-	26.5/14.7
**Ours**	Image	**57.4/35.2**	**42.2/21.4**	**60.2/36.1**	**44.5/23.1**	**63.7/39.5**	**48.1/26.9**
	Video	**27.1/14.3**	11.2/9.1	**29.5/17.71**	**19.6/12.3**	**41.7/24.2**	**30.2/18.1**

**Table 3:** Comparison with SOTA methods on **PSG dataset**. Bold numbers indicate the best results.

Methods	R/mR@20		R/mR@50		R/mR@100	
	PredCls	VSGG	PredCls	VSGG	PredCls	VSGG
IMP [54]	30.5/8.97	17.9/7.35	35.9/10.5	19.5/7.88	38.3/11.3	20.1/8.02
MOTIFS [55]	45.1/19.9	20.9/9.60	50.5/21.5	22.5/10.1	52.5/22.2	23.1/10.3
VCTree [56]	45.9/21.4	21.7/9.68	51.2/23.1	23.3/10.2	53.1/23.8	23.7/10.3
GPSNet [58]	38.8/17.1	18.4/6.52	46.6/20.2	20.0/6.97	50.0/21.3	20.6/7.2
PSG [80]	-	28.2/15.4	-	32.1/20.3	-	35.3/21.5
**Ours**	**52.4/25.6**	**32.9/18.4**	**56.1/28.3**	**35.3/21.6**	**62.7/32.12**	**41.34/23.1**

**Table 4:** Ablation study for **flow–attention direction**. Bold numbers indicate the best results.

Flow–Attention Direction	Modality (X)	R/mR@20		R/mR@50		R/mR@100	
		PredCls	VSGG	PredCls	VSGG	PredCls	VSGG
T ↛ X	Image	32.1/17.4	18.3/9.2	37.15/19.2	21.4/10.5	41.6/21.8	23.5/14.7
	Video	10.2/5.3	4.7/2.4	12.7/7.5	7.1/4.2	16.2/10.8	9.8/7.3
X → T	Image	45.3/23.84	20.4/10.8	48.1/25.6	23.5/12.4	52.8/27.3	29.2/16.1
	Video	15.4/7.1	7.2/4.7	18.3/9.4	9.7/6.2	24.7/12.1	14.1/9.4
T → X	Image	**57.4/35.2**	**42.2/21.4**	**60.2/36.1**	**44.5/23.1**	**63.7/39.5**	**48.1/26.9**
	Video	**27.1/14.3**	**11.2/9.1**	**29.5/17.71**	**19.6/12.3**	**41.7/24.2**	**30.2/18.1**

**Table 5:** Ablation study for **hierarchy awareness**. Bold numbers indicate the best results.

Hierarchy Awareness	Modality (X)	R/mR@20		R/mR@50		R/mR@100	
		PredCls	VSGG	PredCls	VSGG	PredCls	VSGG
✗	Image	46.1/22.3	23.7/12.5	47.2/24.1	24.5/13.4	52.6/28.3	29.8/16.1
	Video	17.5/11.3	10.4/8.7	22.1/14.1	14.1/9.61	27.4/15.2	25.7/16.3
✓	Image	**57.4/35.2**	**42.2/21.4**	**60.2/36.1**	**44.5/23.1**	**63.7/39.5**	**48.1/26.9**
	Video	**27.1/14.3**	**11.2/9.1**	**29.5/17.71**	**19.6/12.3**	**41.7/24.2**	**30.2/18.1**

**Table 6:** Ablation study for **attributes** in our dataset. Bold numbers indicate the best results.

Attributes	R/mR@20		R/mR@50		R/mR@100	
	PredCls	VSGG	PredCls	VSGG	PredCls	VSGG
Appearance	14.5/2.3	1.4/0.3	17.2/6.1	2.3/0.5	21.1/9.1	7.8/2.1
Relationship	13.2/1.8	1.3/0.5	16.4/5.2	2.1/0.4	20.8/8.7	7.5/2.4
Interaction	11.4/1.4	0.9/0.4	14.7/4.1	1.7/0.7	17.7/6.4	6.2/1.6
Position	8.1/0.7	1.5/0.51	10.6/3.7	1.4/0.4	14.2/4.7	4.1/0.9
Situation	5.7/0.2	0.7/0.2	7.2/1.7	0.9/0.3	10.1/2.8	2.8/0.4
All together	**27.1/14.3**	**11.2/9.1**	**29.5/17.71**	**19.6/12.3**	**41.7/24.2**	**30.2/18.1**

## Data Availability

The proposed dataset is available at https://uark-cviu.github.io/GASG/ under the CC-BY 4.0 license to help the research community. Please reach out to the corresponding author for further information.

## Appendix A. Sensor Application’s Correlation with Our Article

Our research leveraged a diverse array of sensor data to enable a comprehensive analysis and understanding. Within the JRDB dataset [81], the authors integrated data streams from various sensors, including RGB cameras and lidars, to capture rich environmental information and facilitate sensor-based applications.

Our annotations primarily focus on pedestrian detection and tracking, utilizing data from multiple sensors to enhance accuracy and robustness. Specifically, we include data streams from RGB cameras positioned in the upper row of the cylindrical stereo camera suite, which capture images for annotation purposes. Additionally, the previous authors utilized point clouds obtained from both the upper and lower 16-channel lidars (Velodynes) for 3D bounding box annotations around pedestrians.

This multi-sensor approach enables us to integrate spatial information from different sources, facilitating the precise localization and tracking of pedestrians in complex environments. By harnessing the complementary strengths of RGB cameras and Lidars, our dataset provides a rich source of annotated sensor data for applications such as pedestrian detection, tracking, and environmental perception.

## Appendix B. GASG Dataset Annotation Pipeline

**GPT4RoI.** Our initial step involves using GPT4RoI to generate textual descriptions corresponding to input bounding boxes. GPT4RoI integrates visual and linguistic data and adeptly handles spatial instructions. During processing, GPT4RoI replaces <$region_{i}$> tags in these instructions with results from RoIAlign, derived directly from the image’s features. This process creates a unique fusion of region-specific data with language embeddings. For an enhanced multimodal understanding, this combination of embeddings is then interpreted via the Vicuna [82] model, a specialized instance of LLaMA [83]. This allows us to input bounding boxes around objects and prompt the system for detailed descriptions, covering aspects like appearance, situation, positioning, interactions, and relationships. For instance, when we input bounding boxes around objects and ask the system questions, such as determining the relationship between individuals in <$region_{1}$> and <$region_{2}$>, the system responds with detailed, context-rich descriptions.

**Post-processing with Spacy.** After generating text with GPT-4RoI, we utilize Spacy v3.0 (https://spacy.io/, accessed on 20 October 2023), a Python library for natural language processing, to refine the text further. We specifically use Spacy to add grammatical tags to each word in the text. This tagging involves identifying the grammatical role of each word and determining whether it is a noun, verb, or adjective, among others. This process is essential for understanding the sentence structure and ensuring that the text is accurate in its content and grammatically coherent.

**Human curation and filtering.** For the final step, we rely on human expertise to ensure the highest quality of our output. Our team carefully reviews the Spacy-processed text using a specially designed filter that helps categorize interactivity types. This human oversight is essential for maintaining the highest standards of accuracy and relevance. It enables us to meticulously confirm and refine the interaction types identified by the LLM, ensuring that our final label is precise.

## Appendix C. Data Format

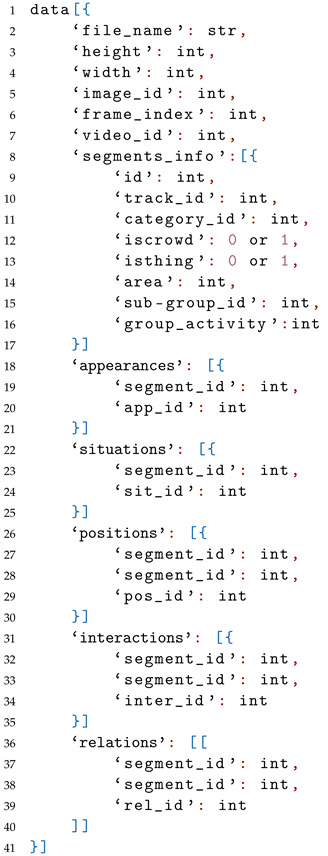

### Appendix C.1. Basic Image Information

This section details the fundamental attributes of each image:

- file_name: The name of the image file.
- height: The height of the image in pixels.
- width: The width of the image in pixels.
- image_id: A unique identifier for the image.
- frame_index: The index of the frame within the video sequence.
- video_id: An identifier for the video or image collection to which this image belongs.

### Appendix C.2. Segment Information

This section includes the segments_info key, which is a list of segments within the image. Each segment contains the following:

- id: A unique identifier for the segment.
- track_id: An identifier to track the segment across different frames.
- category_id: An identifier for the category of the object in the segment.
- iscrowd: A binary value indicating whether the segment represents a crowd.
- isthing: A binary value indicating whether the segment represents a ”thing” (as opposed to ”stuff” like a banner, blanket, curtain, pillow, or towel).
- area: The area covered by the segment in the image.

### Appendix C.3. Interactivity Attributes

This section encompasses lists of predicate_appearances, predicate_situations, predicate_interactions, and predicate_relations for each segment. For single-actor attributes (i.e., appearances and situations), the structure is as follows:

- segment_id: An identifier for the segment.
- id: An identifier for the interactivity type.

For double-actor attributes (i.e., positions, interactions, and relations), the structure includes two different segment_ids to represent the interactivity between two segments:

- segment_id_1: An identifier for the first segment.
- segment_id_2: An identifier for the second segment.
- id: An identifier for the interactivity type.

These descriptors represent lists of integers, specifying various aspects of the subject, object, individual, and group activities for each bounding box within the annotations and segments_info. Please find the complete train and test annotations in the dataset link provided (under Data Availability Statement).

## Appendix D. More Implementation Details

We present the pseudo-code for hierarchy induction in Algorithm A1 and our flow–attention mechanism in Algorithm A2.

**Algorithm A1** Hierarchy induction.		
**Input:** All neighboring affinity scores, $a_{\left(i,j\right),\left(i^{\prime },j^{\prime }\right)}^{l}$, for l={1,⋯,N} layers, a list of “break” threshold values		
	$\left\{\theta_{1},\cdots ,\theta_{N}\right\}$ for every layer.	
1:	l←N	▹ Start from the highest layer
2:	Initialize a nested list, $B=\{B_{1},\cdots ,B_{N}\}$	▹ Store break edges of each layer
3:	**while** l>0 **do**	
4:	**for each** edge $\left(i,j\right),\left(i^{\prime },j^{\prime }\right)$ in the patch graph **do**	
5:	**if** $a_{\left(i,j\right),\left(i^{\prime },j^{\prime }\right)}^{l}<\theta_{l}$ **then**	
6:	**if** l=N **then**	
7:	Append the edge $\left(i,j\right),\left(i^{\prime },j^{\prime }\right)$ to $B_{l}$	▹ Break the edge $\left(i,j\right),\left(i^{\prime },j^{\prime }\right)$ in the top layer *N*
8:	**else**	
9:	**if** edge $\left(i,j\right),\left(i^{\prime },j^{\prime }\right)$ not in $B_{l+1}$ **then**	
10:	Append the edge $\left(i,j\right),\left(i^{\prime },j^{\prime }\right)$ to $B_{l}$	▹ Break the edge $\left(i,j\right),\left(i^{\prime },j^{\prime }\right)$ in layer *l*
11:	**end if**	
12:	**end if**	
13:	**end if**	
14:	**end for**	
15:	l←l−1	▹ Move to the next lower layer
16:	**end while**	
17:	Draw visual hierarchy based on B; then remove redundant edges by finding connected components.	

**Algorithm A2** Multi-head flow–attention mechanism (normal version).
1: **Input:** $Q\in R^{n\times d},K\in R^{m\times d},V\in R^{m\times d}$ 2: Q,K,V=Split(Q),Split(K),Split(V) // $Q\in R^{n\times h\times \frac{d}{h}},K,V\in R^{m\times h\times \frac{d}{h}}$ 3: Q,K=Sigmoid(Q),Sigmoid(K) 4: $I=\mathrm{Sum}\left(Q⊙\mathrm{Broadcast}\left(\mathrm{Sum}(K,\dim=0),\dim=0\right),\dim=2\right)$ // $I\in R^{n\times h}$ 5: $O=\mathrm{Sum}\left(K⊙\mathrm{Broadcast}\left(\mathrm{Sum}(Q,\dim=0),\dim=0\right),\dim=2\right)$ // $O\in R^{m\times h}$ 6: $\hat{I}=\mathrm{Sum}\left(Q⊙\mathrm{Broadcast}\left(\mathrm{Sum}(K/O,\dim=0),\dim=0\right),\dim=2\right)$ // $\hat{I}\in R^{n\times h}$ 7: $\hat{O}=\mathrm{Sum}\left(K⊙\mathrm{Broadcast}\left(\mathrm{Sum}(Q/I,\dim=0),\dim=0\right),\dim=2\right)$ // $\hat{O}\in R^{m\times h}$ 8: $R=\mathrm{Matmul}\left(Q/I,\mathrm{Matmul}\left(K,V⊙\mathrm{Softmax}(\hat{O})\right)\right)⊙\mathrm{Sigmoid}\left(\hat{I}\right)$ // $R\in R^{n\times h\times \frac{d}{h}}$ 9: **Return** R
